# Indigenous knowledge of dye-yielding plants among Bai communities in Dali, Northwest Yunnan, China

**DOI:** 10.1186/s13002-018-0274-z

**Published:** 2018-11-29

**Authors:** Yanxiao Fan, Yanqiang Zhao, Aizhong Liu, Alan Hamilton, Chuanfa Wang, Liangqun Li, Yekun Yang, Lixin Yang

**Affiliations:** 1 Southwest Forestry University, Kunming, 650224 Yunnan China; 2 College of Forestry and Vocational Technology in Yunnan, Kunming, 650224 Yunnan China; 3 Key Laboratory of Economic Plants and Biotechnology, Kunming Institute of Botany, Chinese Academy of Sciences, No. 132 Lanhei Road, Kunming, 650201 China; 4 Center for Biodiversity and Indigenous Knowledge, Kunming, 650034 Yunnan China

**Keywords:** Indigenous knowledge, Dye-yielding plants, Bai communities, Biodiversity protection

## Abstract

**Background:**

Bai people in the Dali Prefecture of Northwest Yunnan, China, have a long history of using plant extracts to dye their traditional costumes and maintain this culture for posterity. However, the development of modern technology, while vastly improving the dyeing efficiency, is also replacing indigenous knowledge which threatens the indigenous practice, causing the latter disappearing gradually. This study sought to examine the indigenous knowledge of plants used for textile dyeing in Bai communities, so as to provide a foundation for their sustainable development.

**Methods:**

We conducted a semi-structured interview among 344 informants (above age 36) selected through a snowball sampling method. Free lists and participant observation were used as supplementary methods for the interviews. Three quantitative indicators (informant consensus factor [ICF], use frequency, and cultural importance index [CI]) were used to evaluate the indigenous knowledge of the dye-yielding plants.

**Results:**

Twenty-three species belonging to 19 plant taxonomic families were used for dye by Bai communities. We summarized them into four life forms, eight used parts, five colors, three processing methods, and four dyeing methods. Among them, *Strobilanthes cusia* (Nees) O. Kuntze was the most traditional dyeing plant and has an important cultural value. Location, age, and gender were found to have a significant effect on indigenous knowledge, and the dyeing knowledge was dynamic and influenced by social factors.

**Conclusions:**

Diverse plant resources and rich indigenous knowledge of textile dyeing persist at settlements of Bai communities in Dali Prefecture. However, high labor costs and thinning market of traditional products that use plant dye cause repulsion toward traditional practice. To that, a good income in other profession attracts indigenous people to shift from their tradition of making plant-based dye and associated cultural systems at risk of extinction. More research for market development for products that use plant-based dye is necessary for the conservation of this valuable knowledge and biodiversity protection in Bai communities.

## Background

Before the discovery of synthetic dyes by William Henry Perkin in 1856, dyes obtained from plants were in use all over the world. Afterward, synthetic dyes, with low cost, plentiful colors, and easy access, gradually replaced natural plant dyes. Nowadays, with increasing realization that some synthetic dyes can be harmful to health and the environment [1–3], there is renewed interest in natural dye-yielding plants [4]. Furthermore, due to the severe threat of ecological globalization, environmental degradation, and cultural homogenization, it is crucial to record the indigenous knowledge of plant utilization and preserve the plants’ habitats, especially where they are not yet completely lost [5].

In China, few ethnobotanical research on dye derived from plants has been carried out among ethnic groups, such as Dai, Buyi, Miao, Yao, Zhuang, Dong, Li, and Weiwuer [6–12]. However, these studies represent only a few ethnic groups of China whose indigenous knowledge is disappearing. Among these ethnic groups, Bai is a special group with abundant indigenous dyeing knowledge, and no research on dyeing plant has been investigated among this group.

With the population of 1.93 million, Bai is the 15th largest ethnic group in China and one of the 25 ethnic groups in Yunnan. In this province, Dali Bai Autonomous Prefecture is home to 58% Chinese Bai population [13]. Historical studies show that the modern Bai ethnic group has emerged from an amalgam of ethnic groups, including the original inhabitants of Kunming, the Heman people around Erhai Lake, the Di people, and Qiang people from Qinghai-Tibetan Plateau. Therefore, traditional Bai culture contains not only cultural elements of Han and Tibetan but also its own characteristics [14, 15]. For example, Bai people often use plant dyes to stain images of camellia flowers, pomegranate fruits, fishes, and butterflies in textile dyeing, which in Bai’s culture are symbols of fertility, wealth, possessions, and good fortune [16–18].

This is the first ethnobotanical study focusing on Bai people’s indigenous knowledge of plant-based dyes. The plant materials, used parts, dyeing methods, and uses are recorded, with data quantified. It provides a foundation for follow-up work to contribute to the maintenance of indigenous plant-dyeing knowledge and dyeing plant resources.

## Methods

### Study sites in Dali Prefecture

Dali Bai Autonomous Prefecture was selected for this research because it is both floristically rich and full of the cultural practice of drawing on biodiversity for dyeing. Located in the middle west of Yunnan Province (24̊41–26̊42′ N, 98̊52–101̊03′ E), on the average, the elevation of this area is 2090 m, annual precipitation received is 776 mm, and annual temperature is 16.5 °C. This study area is the birthplace of the Bai, and likewise where Bai people are most densely settled. It is also regarded as the cultural and linguistic center by this ethnic group [19].

**Fig. 1:**
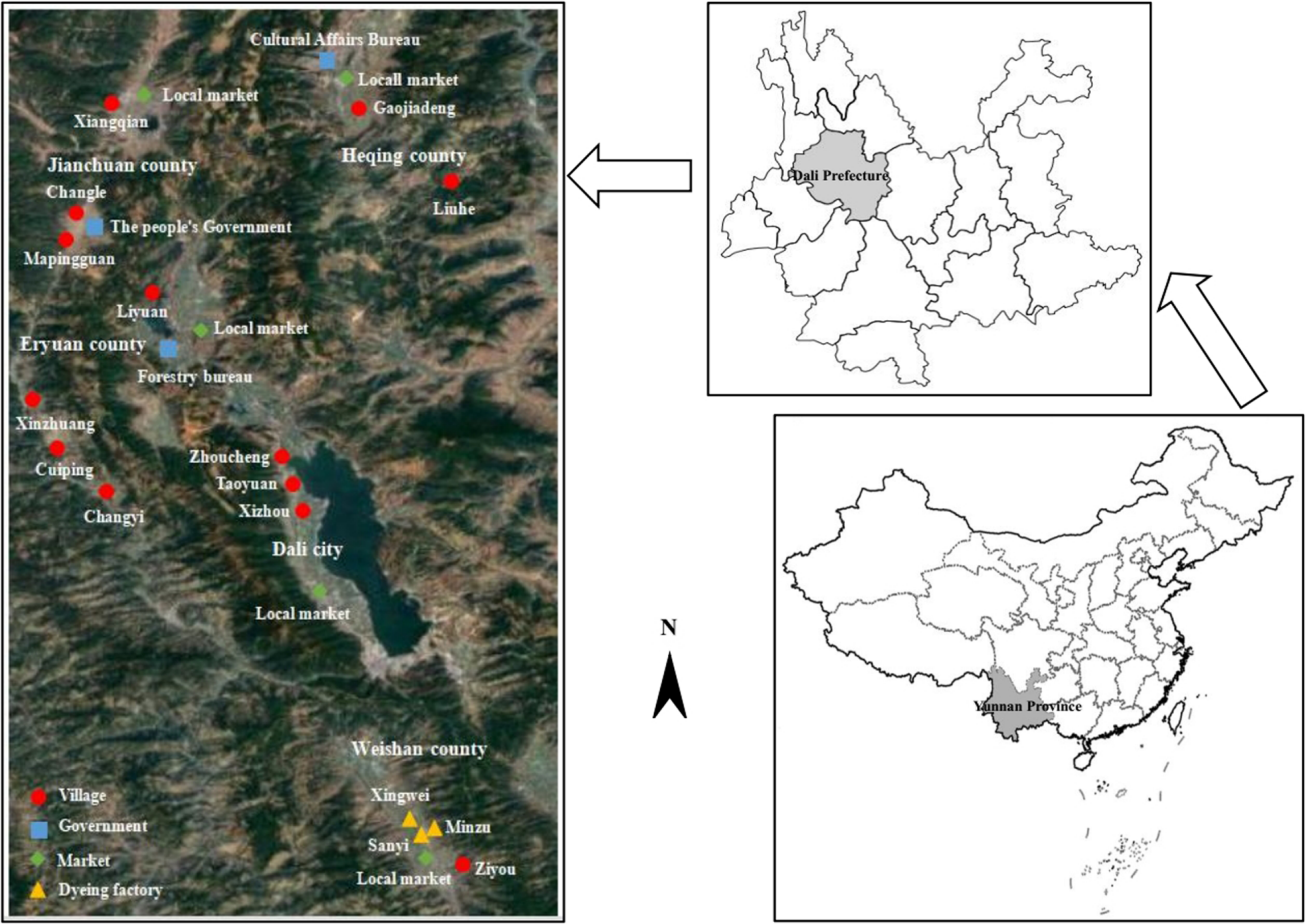
Location map of study sites

After consulting the local government officials and making preliminary field visits, for survey, we selected 13 traditional villages, five local markets, three relevant government agencies, and three local factories that uses plant-based dyes. The survey was conducted in five counties namely Dali, Weishan, Eryuan, Jianchuan, and Heqing which belong to the Prefecture (Fig. 1). Field research was carried out in the study sites between April 2016 and November 2017.

### Ethnobotanical surveys

Prior to this study, locals were contacted and informed about the purpose of the study. Investigations were done based on their consent. Semi-structured interviews were carried out among indigenous people, and questions asked were as follows: (1) Which plants around the communities are useful in deriving natural dye? (2) Which parts of the plant are used? (3) What is the dye used for? (4) What color is the dye? (5) What are the methods of dye? (6) What customs or indigenous knowledge are linked with plant-based dyes? (7) Are there any other uses for these dye-yielding plants?

We used snowball sampling [20] method to select a total of 344 informants that include 197 men and 147 women (Table 1). The informants included farmers, cloth merchants, and indigenous people specialize on plant-based dyes who were aware about plant-based dye and indigenous practice of coloring using plant extracts.

**Table 1:** Demographic details of the informants

Category	Subcategory	Number	% of informants
Location	Weishan	84	25
	Daili	46	13
	Eryuan	73	21
	Jianchuan	71	21
	Heqing	70	20
Age	36–45	21	6
	46–55	58	16
	56–65	114	33
	66–75	108	32
	76–85	43	13
Gender	Male	197	57
	Female	147	43

We tabulated a recorded information about the plants (Table 2) that comprise voucher specimen code, Chinese name, scientific name, local name, family name, life form, used parts, color, main chemical components, and use frequency. We collected 1–2 voucher specimens for each species, adding up to 41 specimens in total, all deposited in the Herbarium, Kunming Institute of Botany, Chinese Academy of Sciences, and verified by the local Bai people, plant taxonomists, and experts from the authors’ institution.

**Table 2:** Dye-yielding plants used by Bai communities in Dali Prefecture

Voucher specimen code	Vernacular name	Scientific name	Local name	Family name	Life form	Used part(s)	Color	Dye use	Main chemical components	Medicinal value	Use frequency (%)
DL02	Yangcong 洋葱	*Allium cepa* Linnaeus	Yangcong	Amaryllidaceae	Herb	Peel	Yellow	Cloth	Phenolic pigments including flavonoids, and flavonols, etc. [27]	Stimulate appetite, help digestion, sterilization, anti-cold, etc.	43
WS08	Chuandian qimu 川滇桤木	*Alnus ferdinandi-coburgii* Schneid.	Haiseizi	Betulaceae	Tree	Bark	Yellow, gray	Cloth	Diaryl heptane compounds, and oleanolic acid, campyloxine, chlorogenic acid, etc. [28]	Liver protection, depressurization, antibacterial, antiviral, anti-inflammatory, diarrhea	92
WS04	Guizhencao 鬼针草	*Bidens pilosa* Linnaeus	Houqizi	Compositae	Herb	Whole Plant (fresh)	Yellow, gray	Cloth	Flavonoids, alkynes, coumarins, organic acids, and its phenols, triterpene, sterols, volatile oils, etc. [29]	Dispersing superficies, clearing away heat, detoxicating, and dissipating stasis	45
HQ02	Mimenghua 密蒙花	*Buddleja officinalis* Maxim.	Suxiuhuo	Loganiaceae	Shrub	Flower	Yellow	Cloth	Flavonoids, including linarin, luteolin, flavonoids, etc. [30]	Expelling wind and cooling blood, moistening liver, improving eyesight, clearing away lung heat, and relieving cough	45
JC03	Sumu 苏木	*Caesalpinia sappan* Linnaeus	Zisu	Leguminosae	Tree	Heartwood	Red	Cloth	Phenolic compounds, flavonoids, and ematoxylin, etc. [31]	Relaxing tendons, activating collaterals, activating blood circulation to dissipate blood stasis and resisting inflammation, etc.	72
WS02	Puercha 普洱茶	*Camellia sinensis* (Linnaeus) Kuntze *var. assamica* (Mast.) Kitamura	Puaizaosei	Theaceae	Tree	Leaves (fresh)	Yellow	Cloth	Chlorophyll, carotenoidl, anthocyanin, anthocyanin, theaflavins, theaflavins, etc. [32]	Antioxidation, anti-aging, reducing blood sugar, blood lipid, preventing cardiovascular system diseases	19
EY01	Honghua 红花	*Carthamus tinctorius* Linnaeus	Hohuo	Compositae	Herb	Flower	Red, yellow	Cloth	Safflower yellow (C_21_H_20_O_10_), safflower red (C_21_H_22_O_11_), flavonoid derivatives, etc. [33]	Activating blood to promote menstruation, dissipate blood stasis, and relieve pain	70
WS06	Huanglian 黄连	*Coptis chinensis* Franch.	Huanglian	Ranunculaceae	Herb	Stem, Root	Yellow	Cloth	Berberine, coptisine, palmatine, worenine, flavonoids and coumarin, etc. [34, 35]	Treat acute conjunctivitis, acute gastroenteritis, hematemesis, and other symptoms of sore carbuncle furuncle	62
EY03	Masang 马桑	*Coriaria nepalensis* WalLinnaeus	Maixiu	Coriariaceae	Shrub	Branches, Leave (dry)	Black, gray, yellow	Cloth	Coriamyrtin, quercetin-3-O-a-L-Arabia sugar, kaempferol-3-O-a-L-Arabia sugar, malol, gallogen, etc. [36]	Clearing away heat and toxic materials, detumescence, pain relieving, and muscle regeneration	93
DL03	Jianghuang 姜黄	*Curcuma longa* Linnaeus	Engexigong	Zingiberaceae	Herb	Root	Yellow	Cloth	Flavonoids, including curcumin, demethoxy curcumin, and bisdemethoxy curcumin, etc. [37, 38]	Anti-mutagenic, anti-tumor, antioxidant, and hypolipidemic	78
DL06	Huangmao qinggang 黄毛青冈	*Cyclobalanopsis delavayi* (Franch.) Schott.	Fusei	Fagaceae	Tree	Bark	Yellow	Cloth	Pedunculagin, astalin castalagin, castavaloninic acid, valolaginic acid, isovalolaginic acid, etc. [39]	Anti-inflammatory, antioxidant, and antibacterial, antiviral	91
DL05	Shizi 柿子	*Diospyros Kaki* Linnaeusf.	Tazi	Ebenaceae	Tree	Fruit	Yellow	Cloth	Lutein, fructose, glucose, sucrose, a variety of vitamins and minerals, especially iodine, etc. [40]	Lowering blood pressure, prevent bleeding hemorrhoids, constipation, suitable for thyroid disease caused by iodine deficiency	46
HQ01	Zijingzelan 紫茎泽兰	*Eupatorium adenophora* Spreng.	Geifacao	Compositae	Herb	Branches, Leaves	Yellow	Cloth	Polysaccharides, anthraquinones, flavonoids,coumarins, and lactones, etc. [41]	Regulating menstruation and activating blood, detoxifying, and eliminating swelling, cure wind-heat type common cold	88
WS09	Zizhihua 栀子花	*Gardenia jasminoides* Ellis	Zizihuo	Rubiaceae	Shrub	Fruit	Yellow	Cloth	Gardenia yellow pigment, saffron, saffron, rutin and xanthophyll, etc. [42]	Clearing away heat, purging fire, cooling blood	77
JC01	Meiguiqie 玫瑰茄	*Hibiscus sabdariffa* Linnaeus	Meiguiqie	Malvaceae	Herb	Fruit	Red	Cloth	Roselle red pigment, delphinidin, scabiolide, and chlorogenic acid, etc. [43]	Clearing away summer heat, eliminate fatigue, lowering blood pressure, relieving asthma, diuresis, detoxification, etc.	27
WS03	Fengxian Hua 凤仙花	*Impatiens balsamina* Linnaeus	Shenjiaihuo	Balsaminaceae	Herb	Flower	Red	Cloth and nails	Anthocyanins, quercetin, lawsone, cyanidinmono-glycoside, etc. [44]	Promoting blood circulation to reduce swelling and treating traumatic injury	83
WS05	Hetao 核桃	*Juglans regia* Linnaeus	Aodao	Juglandaceae	Tree	Peel	Black, yellow, gray	Cloth	Naphthoquinone pigment, and its glycoside, flavonoids, flavonoids, etc. [45]	Anti-mildew, anti-bacterial, anti-tumor	80
JC02	Sang 桑	*Morus alba* Linnaeus	Souwu	Moraceae	Shrub	Fruit	Red	Cloth	Tannic acid, malicacid, carotene, cyanidin, and chrysanthemin, etc. [46]	Improve human immunity, eliminate free radicals, anti-aging	51
EY04	Dianzicao 滇紫草	*Onosma paniculatum* Bur.et Fr.	Zetan	Boraginaceae	Herb	Root	Red	Cloth and nails	Shikonin, Acetyl Shikonin (C_18_ H_18_O_6_), etc. [47]	Hypoglycemic, bacteriostatic, and antitumor	85
DL04	Shiliu 石榴	*Punica granatum* Linnaeus	Ximia	Punicaceae	Shrub	Peel	Yellow	Cloth	Phenolic compounds including ellagic tannins, epigallocatechin, gallic acid, catechins, and anthocyanins, etc. [48]	Antioxidant, antitumor, scavenging oxygen free radicals, and inhibiting atherosclerosis	74
WS07	Mali 麻栎	*Quercus acutissima* Carruth.	Zuilizi	Fagaceae	Tree	Peel	Yellow, gray	Cloth	Tannins, quercetin, flavonoids, anthocyanins, etc. [49]	Antipyretic, astringent, hemostatic, etc.	88
EY02	Qiancao 茜草	*Rubia cordifolia* Linnaeus	Qianhuohuo	Rubiaceae	Liana	Root	Red	Cloth and nails	Anthraquinone derivatives, including alizarin (C_14_H_8_O_4_), alizarin purple pigment (C_14_H_8_O_5_), etc. [50]	Hemostasis, anti-platelet aggregation, elevation of leukocytes, and antitussive sputum	79
WS01 DL01	Banlan 板蓝	*Strobilanthes cusia* (Nees) O. Kuntze	Na	Acanthaceae	Herb	Branches, leaves (fresh)	Blue	Cloth	Indigotine (C16H10N2O3), blue glucoside (C14H17NO6), etc. [51, 52]	Clear heat detoxification, antibacterial antiphlogistic	95

Species in inventory are ordered by the scientific name alphabetically. Vernacular name of dye-yielding plants are written using Chinese pinyin and characters

### Statistical analysis

According to informants, each of the listed plant was used to extract one to three different colors. To determine the consistency of the information, we used the informant consensus factor (ICF) [21] which can be mathematically expressed as:

$$ \mathrm{ICF}=\frac{\mathrm{Nur}-\mathrm{Nt}}{\mathrm{Nur}-1} $$

In this expression, Nur indicates the total number of plant species used to extract a certain color and Nt refers to the number of species simultaneously approved by all informants for dyeing a certain color. ICF values range between 0 and 1, with 0 indicating the highest level of informant consent and 1 the lowest. To quantify the use frequency of certain species [22], the below formula was adopted:

$$ f=\frac{N_m}{N_i} $$

In this formula, *f* represents the use frequency, *N*_*m*_ is the frequency of certain species mentioned by informants, and *N*_*i*_ represents the total number of informants. The higher the value of *f*, the more frequently the plant is used.

Also, the cultural importance index (CI) was calculated to evaluate the cultural significance of dye-yielding plants [23] using the formula below:

$$ \mathrm{C}{\mathrm{I}}_S=\sum \limits_{U={U}_1}^{{}^U\mathrm{NC}}\sum \limits_{i={i}_1}^{{}^iN}\frac{{\mathrm{UR}}_{ui}}{N} $$

*N* is the total number of informants; NC is the total number of usages, for a given dye-yielding plant *S*; UR_*ui*_ represents a utilization report (UR) of species *S* mentioned by the *i*th informant in usage *u*; thus, the CI is the sum of the proportion of informants that mentioned each of the use purpose(s) of a given species. This index indicates the spread of use of each species and the diversity of its uses. Each purpose mentioned is significant to the importance of the plant. Therefore, more usages can result in higher CI value.

## Results

### The diversity of dye-yielding plants in Bai communities

We documented a total of 23 species used by indigenous people to extract different colors that belong to 19 plant families. The majority recorded species were herbaceous (10), while the remaining were trees (7), shrubs (5), and lianas (1). The plant parts used in extracting color include peel (4), fruit (4), leaves (4), roots (4), flowers (3), bark (3), heartwood (1), and the whole plant (1). Yellow (16), red (7), gray (5), black (2), and blue (1) extract were produced from the listed plants (Table 2). All the plants listed in Table 2 were used in textile dyeing process. In addition to that, species like *Impatiens balsamina* L., *Onosma paniculatum* Bur. et Franch., and *Rubia cordifolia* L. were also used for coloring nails. Informants mentioned that these plants were also used by Bai people as source of food, medicine, and ornaments, beside extracting color.

### Indigenous knowledge dynamics in location, age, and gender

Data analysis reveals significant regional differences in the distribution of indigenous knowledge of plant-based dyes. Out of total informants, 25% belong to Weishan, 21% from Eryuan, 21% from Jianchuan, 20% from Heqing, and 13% from Dali (Table 1). Bai people in Dali have the tradition of preparing and selling product colored with plant-based dyes. During survey, we noticed still it was a source of income for local inhabitants, and hence, the culture of using plant-based dye was still alive. Therefore, enough information was obtained by interviewing a few people in Dali. In contrast to Dali, we noticed that Heqing, Jianchuan, Eryuan, and Weishan were highly influenced by the other income-generating opportunities developed along with the local economy; hence, only a few elderly people (especially male), who were knowledgeable, are still using the plant-based dye.

The age difference among the informants was also noticeable: 22% of informants were 36 to 55, 65% were aged 56 to 75, and 13% were 76 and 85. It was clear that older people possessed good knowledge of plant-based dye, while the knowledge decline with younger generation. We noticed that the informants themselves were also aware of this truth. According to them, young people were not willing to get involved in traditional textile dyeing as it is a strenuous activity, and migrating from rural to urban area in search of jobs and different lifestyle.

Moreover, 57% of the informants are male and 43% are female (Table 1); gender role is clearly defined in the traditional textile dyeing profession. According to the informants, men were involved in the collection of plants, the preparation of dyes, and the process of dyeing fabric that requires certain physical strength. Women were mainly responsible for cutting fabrics, designing patterns, and some time-consuming work. As a result, men generally possessed more knowledge about dye-yielding plants and plant collection sites compared to women.

### Evaluation of indigenous knowledge on dye-yielding plants

Three quantitative indicators [ICF (Table 3), *f* (Table 2), and CI (Table 4)] were selected to evaluate 23 species of dye-yielding plants. These indicators showed different ranking of species indicating that the uses of the dye-yielding plants were multiple and the distribution of staining knowledge among informants was dissimilar.

**Table 3:** Informant consensus factor of dye-yielding plants used by Bai people

Color	Total number of plant species (Nur)	The simultaneous utilized number of plant species (Nt)	ICF
Blue	1	1	0.00
Gray	5	4	0.25
Red	7	5	0.33
Yellow	16	11	0.33
Black	2	1	1.00

**Table 4:** Cultural importance index of dye-yielding plants used by Bai people

Scientific name	Use purposes				CI
	Dye	Food	Medicine	Others	
*Strobilanthes cusia* (Nees) O. Kuntze	328	103	337	153 (OR)	2.67
*Punica granatum* L.	253	344	73	213 (OR)	2.57
*Gardenia jasminoides* Ellis	264	25	298	269 (OR)	2.49
*Morus alb*a L.	177	344	275		2.31
*Juglans regi*a L.	274	344	112		2.12
*Diospyros Kaki* L.f.	159	344	57	83 (OR)	1.87
*Cyclobalanopsis delavayi* (Franch.) Schott.	312		261	64 (FI)	1.85
*Curcuma longa* L.	269	46	243	73 (OR)	1.83
*Impatiens balsamina* L.	287		143	174 (OR)	1.76
*Carthamus tinctorius* L.	241		313		1.61
*Coptis chinensis* Franch.	212		317		1.54
*Allium cepa* L.	149	344	9		1.46
*Onosma paniculatum* Bur. et Fr.	291		197		1.42
*Caesalpinia sappan* Linn.	247		211		1.33
*Rubia cordifolia* L.	271		179		1.31
*Hibiscus sabdariffa* Linn.	93	76	274		1.29
*Alnus ferdinandi-coburgii* Schneid.	317		84	41 (FI)	1.28
*Coriaria nepalensis* Wall.	321		39	46 (FI) 23 (FE)	1.25
*Quercus acutissima* Carruth.	301		33	78 (FI)	1.20
*Eupatorium adenophora* Spreng.	301		16	12 (FE)	0.96
*Buddleja officinalis* Maxim.	154	21	47		0.65
*Bidens pilosa* L.	156		27	8 (FE)	0.56
*Camellia sinensis (L.) Kuntze var. assamica (Mast.) Kitamura*	65	39	13	21 (OR)	0.40

Ranked by CI values from high to low

*OR* ornamental, *FI* firewood, *FE* fertilizer, *CI* cultural importance index

#### ICF values

Informants mentioned the use of five different colors, viz. blue, gray, red, yellow, and black. The results of the ICF showed the highest value for black (1), followed by yellow, red, and gray, with values 0.33, 0.33, and 0.25 respectively, while blue had the lowest ICF values (0.00). These distinct ICF values indicated that informants reached different consensus with regard to different color categories. Blue is primarily extracted from *Strobilanthes cusia* (Nees) O. Kuntze, which was widely agreed among informants. Plants that produce black were mainly *Juglans regia* L. and *Coriaria nepalensis* Wall.. *Juglans regia* L. was more common. In addition, the ICF values of the other three categories were less than 0.5, indicating that the plants used for extracting gray, red, and yellow were not very different.

#### Use frequency

The use frequency of *Strobilanthes cusia* (Nees) O. Kuntze, *Impatiens balsamina* L., *Juglans regia* L., *Quercus acutissima* Carruth., *Cyclobalanopsis delavayi* (Franch.) Schott., *Alnus ferdinandi-coburgii* Schneid., *Coriaria nepalensis* Wall., *Onosma paniculatum* Bur. et Franch*.*, and *Eupatorium adenophora* Spreng. were all over 80%, and *Carthamus tinctorius* L., *Coptis chinensis* Franch., *Curcuma longa* L., *Rubia cordifolia* L., *Morus alb*a L., *Punica granatum* L., *Caesalpinia sappan* L., and *Gardenia jasminoides* Ellis were all with use frequency of more than 50%. Some of these plants were harvested from wild in Dali Prefecture, and some were cultivated. The wide use of listed plants did not affect the local ecological settings. *Camellia sinensis* (L.) O. Kuntze var*. assamica* (Mast.) Kitamura, *Bidens pilosa* L., *Allium cepa* L., *Hibiscus sabdariffa* L., *Diospyros kaki* Thunb., and *Buddleja officinalis* Maxim. had a low use frequency (< 50%). According to the informants, the economic and medicinal value of these plants was significantly higher than dye, so Bai people only used these plants to stain some high-grade silk products or some non-selling exhibits.

#### CI values

*Strobilanthes cusia* (Nees) O. Kuntze (2.67), *Punica granatum* L. (2.57), *Gardenia jasminoides* Ellis (2.49), *Morus alba* L. (2.31), *Juglans regia* L. (2.12), *Diospyros kaki*Thunb. (1.87), *Cyclobalanopsis delavayi* (Franch.) Schott. (1.85), *Curcuma longa* L. (1.83), *Impatiens balsamina* L. (1.76), *Carthamus tinctorius* L. (1.61), and *Coptis chinensis* Franch. (1.54) showed higher CI values. High CI values may indicate that the informants were very familiar with the plants and their uses and that the plants had high cultural importance in the community. *Camellia sinensis* (L.) O. Kuntze var*. assamica* (Mast.) Kitamura (0.40), *Bidens pilosa* L. (0.56), *Buddleja officinalis* Maxim. (0.65), and *Eupatorium adenophora* Spreng. (0.96) showed low CI values, which probably means that these plants had few or no other uses besides use in dyeing or that few informants knew other uses. These plants can be of special importance to certain informants.

### Dye-yielding plants and related indigenous knowledge

#### Harvest time

Harvest time of dye-yielding plants depends on the growth cycle. For example, Bai harvest *Strobilanthes cusia* (Nees) O. Kuntze leaves and stems before flowering, usually between August and October, when the pigment content in the plant reaches the highest.

#### Dye-making

**Fig. 2:**
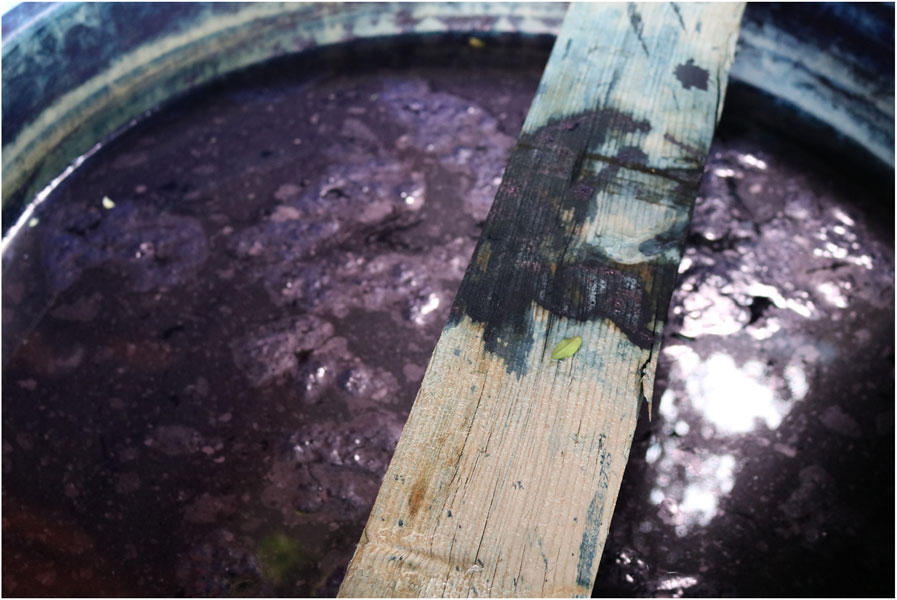
The fermentation of *Strobilanthes cusia* (Nees) O. Kuntze

According to the informants, method varied of dye-making depending upon the source plant material; however, it can be summarized into three main methods: water extraction, fermentation, and mashing. For water extraction, plants were thoroughly washed and useful parts were mashed or cut and then soaked in either hot or cold water for extraction with occasional stir. After (certain time), the mixture was squeezed and filtered to obtain the required solution containing extract. Informants mentioned that the fermentation method is relatively complicated and commonly used in the production of *Strobilanthes cusia* (Nees) O. Kuntze dye. It is necessary to soak the fresh leaves and stems of *Strobilanthes cusia* (Nees) O. Kuntze in the cask to make them ferment, and it is important to keep the leaves and stems remained submerged under water. Sometimes, additional pressure is required to fully dissolve the indigo component in water. Informants mentioned that foam formation on the surface of the water needs to be checked carefully, which means fermentation process is ongoing (Fig. 2). Turning of plant part into red indicated the completion of fermentation process. Mashing is usually used for direct nail coloring.

#### Dyeing method

We documented four different types of dyeing method depending on the mordant used and the order of adding: (1) direct dyeing, which means dyeing without mordant; (2) pre-mordant dyeing where the fiber is soaked in mordant before dyeing; (3) post-mordant dyeing, which entails soaking fiber material with a mordant after plant dyeing; and (4) co-bath dyeing which involves mixing both dyeing mordant and solution together. This method is most popular in Bai communities.

#### Mordant

Informants mentioned that the plant dyes were unstable and easy-fading; therefore, Bai people used some natural mordant to improve their color-fast. Acid mordant (chaenomeles fruit vinegar, black plum juice, lemon juice, etc.), alkaline mordant (plant ash, lime, etc.), and metal mordant (natural minerals) were commonly used. The kind and amount of mordant affect the color of the plant-dyed fabric. For example, *Rubia cordifolia* L. gives the fabric a yellow color when acid mordant chaenomeles fruit vinegar and black plum juice were used to fix the dyes, and red with alkaline mordant. With different mordant quantity, the color varies: small amount induce weak acidity and alkalinity, producing a light color, while large amount generate dark color. Bai people are always known to choose the right type and amount based on their experience to acquire the desired color in dyeing.

### Traditional process of plant-dyeing cloth

**Fig. 3:**
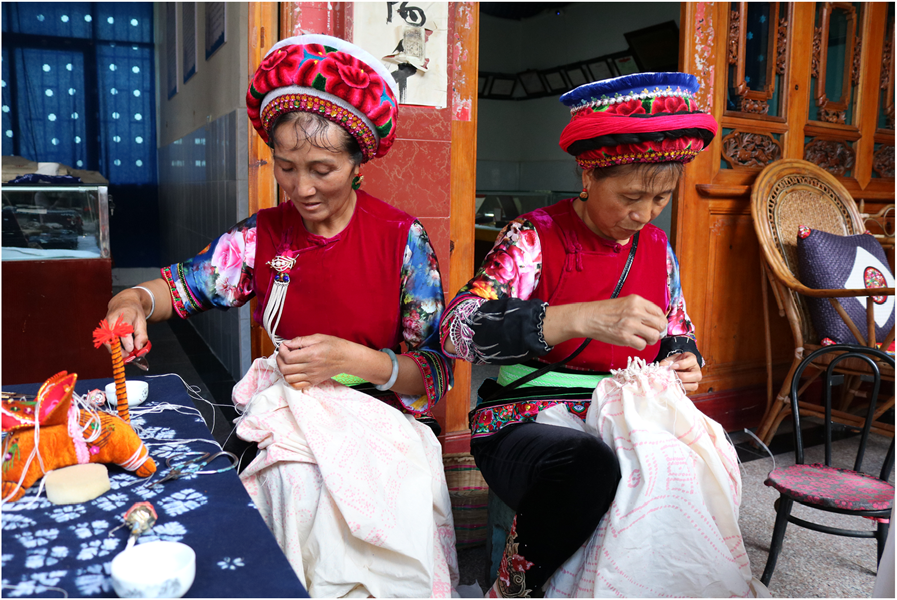
One of the steps of the Bai tie-and-dye *Zahua*

Bai people in Ziyou, Zhoucheng, and Xizhou villages have a unique dyeing technique called tie-and-dye that includes six steps. The first step of tie-and-dye was to design a pattern on a film of plastic. The second was to drill a series of holes along the pattern lines. Later, a cloth was laid under the plastic board, and with a brush dipped with mixed iodine pigment, the board was brushed back and forth. Then, using different needle techniques, they suture the cloth according to the printed pattern. This step is called *Zahua* in Bai language (Fig. 3). Afterward, the tied cloth was repeatedly dip-dyed in *Strobilanthes cusia* (Nees) O. Kuntze dye extract. People using indigenous technique made sure that the cloth got full contact with air to oxidized, so that the pigment get better fixed on the cloth. Finally, after drying the dyed cloth, stitches were removed and washed for the last time for inspecting the final product.

**Fig. 4:**
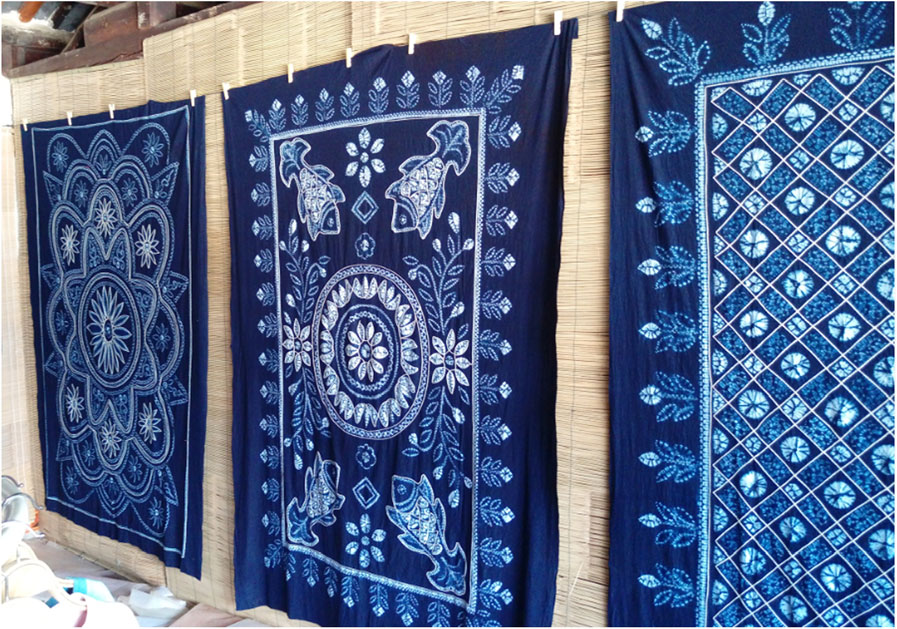
Cloth dyed by *Strobilanthes cusia* (Nees) O. Kuntze

**Fig. 5:**
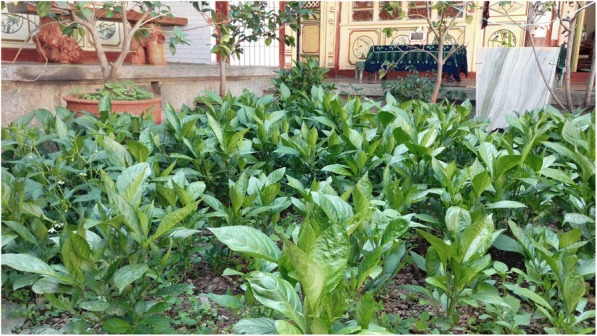
Planting *Strobilanthes cusia* (Nees) O. Kuntze in the yard in Bai communities, Zhoucheng village, Xizhou town, Dali city

*Strobilanthes cusia* (Nees) O. Kuntze (blue dye) was an essential part of tie-and-dye and often mixed with white which we noticed was the favorite color of Bai people; hence, cloths dyed in this way were blue and white (Fig. 4). Due to its importance in traditional culture, Bai people also cultivated *Strobilanthes cusia* (Nees) O. Kuntze in their courtyard (Fig. 5).

### The custom of fingernail plant-dyeing

Every year before their traditional Torch Festival, Bai women use plants to dye their fingernails in bright red. It is mainly for praising women’s quality of loyalty. The custom has persisted since Tang Dynasty (618–907 CE) [24, 25].

Three plant species namely *Rubia cordifolia* L*.*, *Onosma paniculatum* Bur. et Franch., and *Impatiens balsamina* L. were used by Bai people to stain their nails. Species used in different regions were varied. Bai people in Dali and Weishan often dye their nails with the flower of *Impatiens balsamina* L. while in Eryuan, Jianchuan, and Heqing, the roots of *Rubia cordifolia* L. and *Onosma paniculatum* Bur. et Franch. were commonly used. Bai people usually stain their fingernails in spare nights before the Torch Festival. They crushed fresh flower of *Impatiens balsamina* L. or root of *Rubia cordifolia* L. or *Onosma paniculatum* Bur. et Franch. in proper amount of chaenomeles fruit vinegar juice and put the mixture on nails. They then wrap the nails with leaves and wait for the nails to be stained bright red overnight.

### Indigenous knowledge developed in practice

It was observed that indigenous knowledge of plant dyeing developed in the practice. For instance, *Eupatorium adenophora* Spreng. was originally an invasive plant which has brought many adverse effects to the development of local agriculture and animal husbandry. In the course of eradication, the local people accidentally found that *Eupatorium adenophora* Spreng. juice stained the skin and clothes that cannot be removed easily. Therefore, Bai indigene, with rich plant-dyeing experience, began to explore *Eupatorium adenophora* Spreng. as a potential dye, exploring its value and reducing its harm through continuous practice. Extraction of pigments from fresh stems and leaves of *Eupatorium adenophora* Spreng. with boiling water and dyeing after filtration was selected to be the most effective staining method. Nowadays, local dyeing factories in Dali had industrialized the application of *Eupatorium adenophora* Spreng. Bai people used their indigenous knowledge to turn harmful invasive plants into valuable ones.

## Discussion

Out of 23 dye-yielding plants used by Bai, *Strobilanthes cusia* (Nees) O. Kuntze is well known among the local people due to the tradition of tie-and-dye and already industrialized in as early as the Republic of China (1912–1949) [26]. In contrast to that, *Eupatorium adenophora* Spreng., *Coriaria nepalensis* Wall., *Alnus ferdinandi-coburgii* Schneid., *Cyclobalanopsis delavayi* (Franch.) Schott., and *Quercus acutissima* Carruth. are for the first time recorded as dye-yielding plant in Bai communities of Dali Prefecture. The plants used are diverse and are constantly enriched in dyeing practice among Bai communities.

However, the inheritance of indigenous knowledge of plant-based dye is facing some challenges because many of the knowledgeable person in dye processing in Bai communities are aged and chemical dyes are gradually taking the place of plant dyes. At the same time, high labor cost of plant dyes and low production plant dyes are placed onto a disadvantageous position in its competition with chemical dyes. Besides, economic development brings more working opportunities in other professions, which attracts local people to shift from traditional careers to new ones, risking the popularity of traditional plant-dyeing craftsmanship and its supporting cultural system.

To retain the tradition of plant-based dye, local dyeing factories in Dali Prefecture began to incorporate some plant used on small scale into industrial production. After continuous attempts, the dyeing processes of *Eupatorium adenophora* Spreng., *Coriaria nepalensis* Wall., *Alnus ferdinandi-coburgii* Schneid., *Cyclobalanopsis delavayi* (Franch.) Schott., and *Quercus acutissima* Carruth. became developed and have attained initial commercialization in the local dyeing factories. During field survey, the factories that we studied are Xingwei Art & Crafts factory (Xingwei), Sanyi Tie-and-Dye Industry & Commercial Co. Ltd. (Sanyi), and Minzu Craft Clothing factory (Minzu).

Taking Xingwei as an example, a family operates the factory, and staff are locally recruited, who are knowledgeable on traditional tie-and-dye technology. According to informants, plant-based dyes are used in a few products; in 2016, up to 30% of total product was based on plant dyes. Products range from clothing to curtains, cushion cases, hats, handkerchiefs, napkin pads, and so on. Among these products, plant dyed ones are often sold to high-end product markets in Hongkong, Beijing, and Shanghai and exported to Japan, Korea, UK, and so on, while chemical dyed products are usually sold to domestic mass market.

Some limiting factors for commercialization of the plant dyes were identified after discussion at factories and informants. The main disadvantages are (1) the color fastness, which involves instability of dye, causing easy discoloration under conditions of water immersion, sweat stain, and sun exposure; (2) the production cost is higher than chemical dyeing because it is time consuming and labor intensive; and (3) the industrialized application of dye-yielding plants requires a large amount of raw material, which cannot be met solely by wild collection.

Regarding these issues, it is suggested that local governments should provide support for the research institutes as well as local universities to study the characteristics of different kinds of dyeing plants in practice, develop environment-friendly mordant to improve the color fastness of plant dyes, simplify dyeing method, reduce production cost, and adapt to mass production. Besides, it is crucial to establish their own ecological dyeing brand to accelerate the industrialization process and facilitate plant dyed products for a bigger market. Furthermore, we suggest formulation of policies (government and non-governmental organizations) to promote the supply of raw materials through local artificial cultivation and base planting.

## Conclusion

As a traditional ethnic group that uses plant for dye, Bai in Dali Prefecture, Northwest Yunnan, China, owns unique knowledge on plant-based dye and has creatively developed many plant dyes. Our research documents 23 dye-yielding plant species belonging to 19 taxonomic families and selects three quantitative indicators—ICF, *f*, and CI—to evaluate the documented species. The findings highlight Bai’s indigenous knowledge and practice of dyeing which includes three kinds of common plant dye making methods (water extraction, fermentation, and mashing), four dyeing methods (direct dyeing, pre-mordant dyeing, post-mordant dyeing, and co-bath dyeing), and tie-and-dye, the traditional craft of Bai people.

In summary, this study provides a deep understanding of Bai indigenous knowledge of dye-yielding plants. From the study, it can be concluded that there is need to in-depth research for the stability, safety, and functional ingredients of dye-yielding plants. Same time awareness about traditional importance of traditional textile dyeing, market-oriented research, and opportunity created for products that uses plant-based dye can support to conserve the traditional knowledge of plant-based dye and associated culture.
